# Supplementary material for: Early Embryo Exposure to Assisted Reproductive Manipulation Induced Subtle Changes in Liver Epigenetics with No Apparent Negative Health Consequences in Rabbit
Source: Int J Mol Sci. 2021 Sep 8;22(18):9716. doi: 10.3390/ijms22189716 (PMC8467347; doi:10.3390/ijms22189716)
Supplement: Supplementary file 1 [file ijms-22-09716-s001.zip › Supplementary Figure S1.pdf]

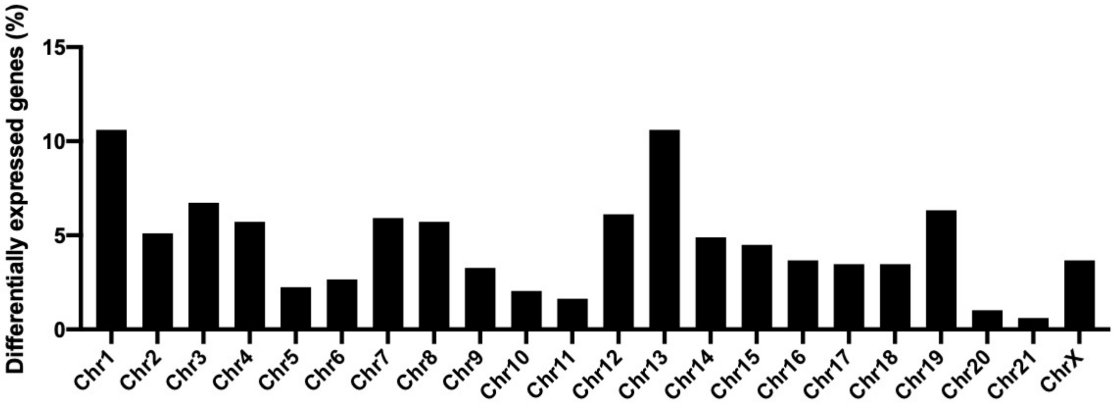

Supplementary Figure S1. Percentage of differentially expressed genes of our previous transcriptomic study that belong from each chromosome. [16] Garcia-Dominguez, X.; Marco-Jiménez, F.; Peñaranda, D.S.; Diretto, G.; García-Carpintero, V.; Cañizares, J.; Vicente, J.S. Long-term and transgenerational phenotypic, transcriptional and metabolic effects in rabbit males born following vitrified embryo transfer. Sci. Rep. 2020, 10, doi:10.1038/s41598-020-68195-9.
