# Supplementary material for: Early Embryo Exposure to Assisted Reproductive Manipulation Induced Subtle Changes in Liver Epigenetics with No Apparent Negative Health Consequences in Rabbit
Source: Int J Mol Sci. 2021 Sep 8;22(18):9716. doi: 10.3390/ijms22189716 (PMC8467347; doi:10.3390/ijms22189716)
Supplement: Supplementary file 1 [file ijms-22-09716-s001.zip › Supplementary Table S1.pdf]

Supplementary Table S1. Targeted identification of differentially accumulated metabolites in liver tissue of F3 generation animals derived from early embryos exposed to the combination of multiple ovulations, vitrification and embryo transfer (MOVET) and naturally-conceived (NC). The negative values are down-accumulated metabolites in the MOVET group, while the positive are up-accumulated.

Metabolic pathway	Metabolite name	Fold change (Log2 MOVET/NC)
Biosynthesis of amino acids	Arginine	0.13
	Asparagine	0.26
	Aspartate	0.28
	Cysteine	**-2.00**
	Glutamic_acid	0.29
	Glutamine	-0.15
	Histidine	**0.23**
	Isoleucine-leucine	0.46
	Lysine	0.05
	Methionine	0.27
	Phenylalanine	0.24
	Proline	0.28
	Serine	-1.65
	Threonine	0.31
	Tryptophan	**-1.07**
	Tyrosine	0.35
	Valine	0.72
Arachidonic acid metabolism<br>Linoleic acid metabolism	11-dehydro_Thromboxane_B2	0.12
	Hepoxilin_A3	0.32
	Leukotriene_F4	0.17
	Prostaglandin_B2	**-0.38**
	Prostacyclin	0.08
	11,14,15-theta	0.39
	15-Keto-prostaglandin_f2alpha	**-0.52**
	6-ketoprostaglandin_e1	0.17
	Trioxilin_A3	0.18
	11,14,15-theta	-0.34
	5,6-Dihydroxy-8Z,11Z,14Z-eicosatrienoic_acid	0.24
	5-hete	**-0.47**
	5-oxo-ete	**-0.38**
	6-ketoprostaglandin_e1	-0.16
	Lecithins	**-1.15**
	Tetrahydro-3,4-furandiol	-0.10
	Prostacyclin	-0.38
	14,15,19-/14,15,20-trihydroxy-5,8,11-eicosatri	**-0.42**
	11(12)oxido-5,8,14-eicosatrienoic_acid	**-0.52**

	14,15-dihydroxy-5,-8,11-eicosatrienoic_acid	**-0.60**
	11,12,19-/11,12,20-trihydroxy-5,8,14-eicosatri	**-0.53**
	5(6)oxido-8,11,14-eicosatrienoic_acid	**-0.86**
	PC_(36:5)	**-0.93**
	PC_(38:7)	-0.15
	PC_(34:4)	**-0.75**
	PC_(30:3)	**-0.65**
	PC_(40:6)	-0.15
	PC_(38:6)	-0.39
	PC_(36:4)	**-0.76**
	PC_(30:0)	**-0.82**
	PC_(34:1)	-0.36
	PC_(34:2)	**-0.61**
	PC_(38:5)	**-0.61**
	PC_(36:3)	**-0.61**
	PC_(38:4)	**-0.62**
	PC_(34:0)	**-0.45**
	PC_(32:1)	**-0.65**
	PC_(36:1)	**-0.73**
	PC_(32:0)	-0.30
	PC_(36:2)	**-0.81**
	PC_(40:5)	**-0.91**
	PC_(34:6)	**-0.82**
Glycolisis Gluconeogenesis	2-hydroxy-ethyl-thpp	0.65
	Camp	0.47
	Glucose	-0.41
	Glyceraldehyde-3P	0.14
	Glycerate-1,3P2	-0.21
	Glycerate-3P	**2.57**
	ß-D-Fructose-1,6P2	**-0.44**
	ß-D-Glucose-6P	0.15
	Thpp	**1.65**
	S-acetyl-dihydrolipoamide-e	**0.34**
	Lipoamide-E	-0.34
Oxidative phosphorylation	Adenosine_diphosphate	0.05
	Fad2+	-0.13
	Fumarate	0.41
	Gluconate	-0.15
	Glucose	-0.41
	Nadh	1.05
	Succinate	0.02
Biosynthesis of unsaturated fatty acids	Adrenic_acid	**-0.77**

	Arachidic_acid	**-0.48**
	Arachidonic_acid	**-0.58**
	Behenic_acid	-0.61
	Docosadienoic_acid	**-0.49**
	Docosahexaenoic_acid	**-0.36**
	Docosapentaenoic_acid	**-0.71**
	Erucic_acid	**-0.17**
	Icosadienoic_acid	**-0.18**
	Icosapentaenoic_acid	**-0.35**
	Icosatrienoic_acid	**-0.32**
	Icosenoic_acid	-0.10
	Lignoceric_acid	-0.42
	Linoleic_acid	**-0.67**
	Nervonic_acid	**-0.63**
	Oleic_acid	**-0.48**
	A-Linolenic_acid	**-0.58**
	Palmitic_acid	**-0.28**
	Stearic_acid	**-0.20**
Steroid biosynthesis	24-epi-Campesterol	**-0.70**
	24-methylenecholesterol	2.87
	5-dehydroavenasterol	0.18
	7-dehydrocholesterol	-0.16
	7alpha,24-Dihydroxy-4-cholesten-3-one	**-0.44**
	Episterol	**-0.34**
	Lathosterol	-0.26
	Secalciferol	-0.79
	3alpha,7alpha-Dihydroxy-5beta-cholestanate	-0.04
	S-squalene_2,3-epoxide	**-0.56**
	22(r)-hydroxycholesterol	**-0.98**
	3alpha,7alpha,26-Trihydroxy-5beta-cholestan	-0.10
	Calcidiol	-0.06
	14-demethyllanosterol	0.01
	4alpha-Methylzymosterol-4-carboxylate	0.92
	Squalene	**-0.54**
	3alpha,7alpha,12alpha,26-Tetrahydroxy-5beta	0.80
	Calcitriol	-0.62
	Cholesterol	**-0.14**
	7-dehydrodesmosterol	**-0.99**
	Campesterol	-0.10
	3alpha,7alpha-Dihydroxy-5beta-cholestane	**-0.29**
	Pregnenolone	-0.66
	Calcitetrol	0.90

	4alpha-Methylzymosterol	-0.52
Steroid hormone biosynthesis	Cortisol	-0.30
	6 β hydroxy testosterone	0.07
	Aldosterone	**-0.52**
	Androsterone	-0.14
	B-Estradiol	-0.20
	Corticosterone	0.35
	Cortol	-0.37
	Cortolone	-0.45
	Estriol	**-0.19**
	Methoxyestrone	0.01
	Pregnenolone	0.28
	Progesterone	-0.13
	Testosterone	**-0.43**
	Cortol	-0.42
	Pregnanediol	-0.49
Glycerolipid metabolism	TAG_(50:1)	**-1.86**
	TAG_(50:4)	**-1.98**
	TAG_(54:5)	**-1.06**
	TAG_(54:9)	**-1.36**
	TAG_(54:8)	**-1.59**
	TAG_(52:1)	**-1.17**
	TAG_(52:2)	**-1.11**
	TAG_(50:0)	**-1.38**
Citrate cycle (TCA cycle)	2-hydroxy-ethyl-thpp	0.65
	3-carboxy-1-hydroxypropyl-thpp	-0.85
	Cis-aconitate	0.10
	Citrate	**-0.30**
	Oxalosuccinate	**2.09**
	Succinate	0.02
	Thpp	**1.65**
	S-acetyl-dihydrolipoamide-e	**0.34**
	Lipoamide-E	-0.34

Red denotes statistical differences at $p<0.05$.
