# Supplementary material for: Early Embryo Exposure to Assisted Reproductive Manipulation Induced Subtle Changes in Liver Epigenetics with No Apparent Negative Health Consequences in Rabbit
Source: Int J Mol Sci. 2021 Sep 8;22(18):9716. doi: 10.3390/ijms22189716 (PMC8467347; doi:10.3390/ijms22189716)
Supplement: Supplementary file 1 [file ijms-22-09716-s001.zip › Supplementary Table S2.pdf]

Supplementary Table S2. Differentially expressed proteins in liver tissue of F3 generation animals derived from early embryos exposed to the combination of multiple ovulations, vitrification and embryo transfer (MOVET) and naturally-conceived (NC). The negative values are down-expressed proteins in the MOVET group, while the positive are up-expressed.

Uniprot accession	Gene name	Fold change (Log2 MOVET/NC)
U3KPP4	Cytochrome P450 2C30	-3.78
G1T1G8	Periplakin	-3.60
G1SRH7	Serine and arginine rich splicing factor 3	-2.08
G1TYL5	2'-deoxynucleoside 5'-phosphate N-hydrolase 1	-1.95
A0A0A0MQQ2	S100 calcium binding protein A12	-1.95
G1TN25	Alpha-2-glycoprotein 1, zinc-binding	-1.91
G1T7F1	Histone H1.4	-1.66
G1TIS5	Annexin A1	-1.62
P12345	Glutamic-oxaloacetic transaminase 2	-1.55
G1SJS1	Histone H2B type 2-E	-1.37
G1U522	Protein kinase camp-dependent type II regulatory subunit alpha	-1.27
G1SMM7	Small nuclear ribonucleoprotein D3 polypeptide	-1.21
G1TC61	Putative RNA-binding protein Luc7-like 2	-1.07
G1T7S1	Aflatoxin B1 aldehyde reductase member 3	-1.04
G1TUX5	Protein HP-25 homolog 2	-1.01
G1T5K6	Translocase of outer mitochondrial membrane 40 like	-0.97
G1TP83	Histone H2B type 1	-0.94
B7NZF9	Nucleophosmin	-0.93
G1TN62	40S ribosomal protein S19	-0.89
G1TWP4	Valyl-trna synthetase	-0.84
G1T726	Hydroxyacyl-coa dehydrogenase	-0.83
G1TFX2	Alpha-1-antitrypsin	-0.83
G1SHI0	Acetyl-coa acyltransferase 1	-0.81
G1SSN2	Sirtuin 5	-0.71
G1SN67	Serpin family B member 1	-0.67
G1U7L4	Heat shock protein family A	-0.59
G1T9I4	Sorcin	-0.57
G1TBU9	Acetyl-coa acyltransferase 2	-0.51
G1SU01	Nipsnap homolog 3A	-0.43
G1TES6	Hydroxysteroid 17-beta dehydrogenase 10	-0.40
G1T3Y8	Heat shock protein family D	-0.27
G1TU13	40S ribosomal protein S17	-0.23
G1T0L9	Ribophorin I	-0.13
G1TPR2	Glucosamine-phosphate N-acetyltransferase 1	0.30
G1SVP7	Glutathione S-transferase omega 1	0.30
G1TE35	Nudix hydrolase 12	0.35

G1T3Z1	Mannose binding lectin 2	0.40
G1SVJ1	Dihydropyrimidine dehydrogenase	0.42
G1TS42	Amylo-alpha-1, 6-glucosidase, 4-alpha-glucanotransferase	0.45
G1SV60	Transmembrane and coiled-coil domains 1	0.46
G1SN14	Cullin associated and neddylation dissociated 1	0.48
G1TAH7	Transketolase	0.49
G1U2K8	Cytochrome P450-like	0.51
G1TCT8	Major facilitator superfamily domain containing 9	0.53
G1T6X2	Cytochrome P450 2C15-like	0.54
G1U7D9	Solute carrier family 27 member 5	0.57
U3KLZ1	Flavin containing monooxygenase 3	0.66
G1SMY3	2-acylglycerol O-acyltransferase 2-B	0.67
G1TBR1	NAD	0.68
G1T3H9	Desmocollin 2	0.71
G1U4I6	Fatty acid amide hydrolase	0.80
G1SKJ7	Glycine N-methyltransferase	0.80
G1TUC2	CCHC-type zinc finger nucleic acid binding protein	0.82
G1TZE5	Chromosome 13 open reading frame, human c1orf50	0.96
G1TYM3	Cytochrome P450, family 2, subfamily b, polypeptide 4	0.96
G1TLX2	Cytochrome P450 2C16	0.98
G1T9T6	Ethanolamine-phosphate phospho-lyase	1.01
G1T932	Pipecolic acid and sarcosine oxidase	1.03
G1TI39	Radixin	1.03
G1SL36	Glycine decarboxylase	1.04
G1TR70	Cytochrome P450 2C4	1.07
U3KM06	UDP-glucuronosyltransferase 2A3	1.14
G1U9S0	Cytochrome P450 2C2	1.20
G1T0Z2	Histone H2A type 1-A	1.51
G1SUM7	ATP binding cassette subfamily C member 2	1.66
G1SEE9	Thyroid hormone responsive	1.76
G1TGH4	Retinol saturase	1.77
G1TPC5	Tumor protein D52	1.79
G1ST24	Acetyl-coa carboxylase beta	2.05
G1TTZ8	Formimidoyltransferase cyclodeaminase	2.13
G1SZ66	Mannose-P-dolichol utilization defect 1	5.80
