# Supplementary material for: Early Embryo Exposure to Assisted Reproductive Manipulation Induced Subtle Changes in Liver Epigenetics with No Apparent Negative Health Consequences in Rabbit
Source: Int J Mol Sci. 2021 Sep 8;22(18):9716. doi: 10.3390/ijms22189716 (PMC8467347; doi:10.3390/ijms22189716)
Supplement: Supplementary file 1 [file ijms-22-09716-s001.zip › Supplementary Table S3.pdf]

Supplementary Table S3. Functional analysis of differentially expressed proteins in liver tissue of F3 generation animals derived from early embryos exposed to the combination of multiple ovulations, vitrification and embryo transfer (MOVET) and naturally-conceived (NC).

Category*	Term	Count	p-value
BP	Defense response to Gram-positive bacterium	3	0,01
BP	Negative regulation of ryanodine-sensitive calcium-release channel activity	2	0,02
BP	Very long-chain fatty acid metabolic process	2	0,03
BP	Negative regulation of catalytic activity	2	0,04
BP	Positive regulation of G1/S transition of mitotic cell cycle	2	0,05
BP	Innate immune response in mucosa	2	0,07
BP	Antibacterial humoral response	2	0,07
CC	Organelle membrane	7	0,00
CC	Endoplasmic reticulum membrane	10	0,00
CC	Extracellular exosome	23	0,00
CC	Nucleosome	4	0,00
CC	Myelin sheath	5	0,00
CC	Mitochondrial inner membrane	5	0,00
CC	Extracellular space	9	0,01
CC	Integral component of endoplasmic reticulum membrane	3	0,02
CC	Peroxisome	3	0,02
CC	Cytosol	8	0,04
CC	U1 snrnp	2	0,04
MF	Aromatase activity	6	0,00
MF	Heme binding	7	0,00
MF	Iron ion binding	7	0,00
MF	Monooxygenase activity	4	0,00
MF	Oxidoreductase activity, acting on paired donors, with incorporation or reduction of molecular oxygen	4	0,00
MF	Transferase activity, transferring acyl groups other than amino-acyl groups	3	0,00
MF	Oxidoreductase activity, acting on paired donors, with incorporation or reduction of molecular oxygen, reduced flavin or flavoprotein as one donor, and incorporation of one atom of oxygen	3	0,00
MF	Folic acid binding	2	0,03
MF	3-hydroxyacyl-coa dehydrogenase activity	2	0,04
MF	NAD+ binding	2	0,06
MF	Poly(A) RNA binding	9	0,07
KEGG	Retinol metabolism	8	0,00
KEGG	Chemical carcinogenesis	8	0,00
KEGG	Metabolic pathways	22	0,00
KEGG	Steroid hormone biosynthesis	7	0,00
KEGG	Linoleic acid metabolism	5	0,00

KEGG	Arachidonic acid metabolism	6	0,00
KEGG	Biosynthesis of antibiotics	7	0,00
KEGG	Inflammatory mediator regulation of TRP channels	5	0,00
KEGG	Serotonergic synapse	5	0,01
KEGG	Valine, leucine and isoleucine degradation	4	0,01
KEGG	Systemic lupus erythematosus	5	0,01
KEGG	Fatty acid degradation	3	0,04
KEGG	Glycine, serine and threonine metabolism	3	0,04
KEGG	Fatty acid metabolism	3	0,05
KEGG	Drug metabolism - cytochrome P450	3	0,10

*Functional analysis was referred to the GO term annotation according to the biological process (BP), cellular component (CC) and molecular function (MF) classification, and the KEGG pathways in which they are involved. Red denotes statistical differences at $p<0.05$.
