# Supplementary material for: Early Embryo Exposure to Assisted Reproductive Manipulation Induced Subtle Changes in Liver Epigenetics with No Apparent Negative Health Consequences in Rabbit
Source: Int J Mol Sci. 2021 Sep 8;22(18):9716. doi: 10.3390/ijms22189716 (PMC8467347; doi:10.3390/ijms22189716)
Supplement: Supplementary file 1 [file ijms-22-09716-s001.zip › Supplementary Table S4.pdf]

Supplementary Table S4. Differentially methylated genes in liver tissue of F3 generation animals derived from early embryos exposed to the combination of multiple ovulations, vitrification and embryo transfer (MOVET) and naturally-conceived (NC). The negative values are hypomethylated genes in the MOVET group, while the positive are hypermethylated.

Type	Gene accession	Gene name	Chromosome /scaffold	Δβ (MOVET-NC)
Hypo	ENSOCUG00000023366	WD repeat domain 4	GL019261	-4.70
Hypo	ENSOCUG00000017543	Acylglycerol kinase, mitochondrial	GL019263	-3.67
Hypo	ENSOCUG00000003231	TBC1 domain family member 14	GL019275	-3.61
Hypo	ENSOCUG00000007282	SVOP like	GL018853	-3.14
Hypo	ENSOCUG00000004404	Multiple C2 and transmembrane domain containing 2	GL018708	-3.05
Hypo	ENSOCUG00000006366	S100 calcium binding protein P	GL019770	-3.05
Hypo	ENSOCUG00000026584	Ras association domain-containing protein 3	GL018886	-2.91
Hypo	ENSOCUG00000015993	Par-6 family cell polarity regulator beta	GL018822	-2.81
Hypo	ENSOCUG00000004266	Family with sequence similarity 222 member A	GL019210	-2.68
Hypo	ENSOCUG00000023160	Olfactory receptor 6B3		-2.65
Hypo	ENSOCUG00000007918	NOP2/Sun RNA methyltransferase family member 2	GL018756	-2.51
Hypo	ENSOCUG00000007288	Lon peptidase 2, peroxisomal	GL018732	-2.51
Hypo	ENSOCUG00000002796	DDB1- and CUL4-associated factor 12-like protein 2		-2.50
Hypo	ENSOCUG00000013731	Runt related transcription factor 1	GL018729	-2.48
Hypo	ENSOCUG00000014651	Dexh-box helicase 9	GL019071	-2.42
Hypo	ENSOCUG00000004927	Breast carcinoma amplified sequence 1	GL018712	-2.33
Hypo	ENSOCUG00000007274	Tripartite motif containing 24	GL018853	-2.28
Hypo	ENSOCUG00000014336	Multivesicular body subunit 12B	GL018699	-2.27
Hypo	ENSOCUG00000009223	Docking protein 5	GL018712	-2.26
Hypo	ENSOCUG00000016405	Evc ciliary complex subunit 2	GL018874	-2.18
Hypo	ENSOCUG00000009429	1-acylglycerol-3-phosphate O-acyltransferase 5	GL018713	-2.03
Hypo	ENSOCUG00000010151	WD repeat and FYVE domain containing 2	GL018705	-2.01
Hypo	ENSOCUG00000007803	Disco interacting protein 2 homolog C	GL018778	-1.98
Hypo	ENSOCUG00000004466	WD repeat domain 37	GL018707	-1.93
Hypo	ENSOCUG00000023551	Dnaj homolog subfamily C member 8	GL018701	-1.91
Hypo	ENSOCUG00000008722	KIAA2022 ortholog	GL018757	-1.63
Hypo	ENSOCUG00000003497	SHC binding and spindle associated 1 like	GL019071	-1.45
Hypo	ENSOCUG00000000876	Integrator complex subunit 6	GL018705	-1.33
Hypo	ENSOCUG00000016847	ATP binding cassette subfamily B member 7	GL018757	-1.25
Hypo	ENSOCUG00000013902	Sperm acrosome associated 4	GL019039	-0.94
Hypo	ENSOCUG00000016120	Nucleolar GTP-binding protein 1	GL018778	-0.70

Hypo	ENSOCUG00000005446	ADP ribosylation factor guanine nucleotide exchange factor 2	GL018725	-0.61
Hypo	ENSOCUG00000017426	Glutamate metabotropic receptor 7	GL018703	-0.60
Hyper	ENSOCUG00000001208	Relaxin/insulin like family peptide receptor 1	GL018701	0.84
Hyper	ENSOCUG00000005566	G protein-coupled receptor 50	GL018731	1.11
Hyper	ENSOCUG00000012148	Inositol 1,4,5-trisphosphate receptor type 1	GL018703	1.21
Hyper	ENSOCUG00000015945	Mutated in colorectal cancers	GL018744	1.25
Hyper	ENSOCUG00000003938	Deleted in lymphocytic leukemia, 7	GL018705	1.26
Hyper	ENSOCUG00000001660	Von Willebrand factor C and EGF domains	GL018717	1.29
Hyper	ENSOCUG00000017773	Tankyrase	GL018709	1.35
Hyper	ENSOCUG00000021608	Pyridoxal	GL019091	1.43
Hyper	ENSOCUG00000004645	Whirlin	GL018699	1.44
Hyper	ENSOCUG00000003760	Kelch like family member 2	GL018701	1.46
Hyper	ENSOCUG00000001573	Protein tyrosine phosphatase, receptor type T	GL018718	1.50
Hyper	ENSOCUG00000001518	LCA5L, lebercilin like	GL018907	1.51
Hyper	ENSOCUG00000005132	Williams-Beuren syndrome chromosome region 28	GL018765	1.53
Hyper	ENSOCUG00000021767	Hydroxylysine kinase	GL018746	1.53
Hyper	ENSOCUG00000001499	Pleckstrin homology domain containing A4	GL019028	1.55
Hyper	ENSOCUG00000006423	BCL2 associated X, apoptosis regulator	GL019028	1.55
Hyper	ENSOCUG00000002815	Interferon gamma receptor 2	GL018729	1.60
Hyper	ENSOCUG00000009375	Protein phosphatase 6 catalytic subunit	GL018699	1.62
Hyper	ENSOCUG00000004344	Forkhead box K1	GL018871	1.65
Hyper	ENSOCUG00000012302	PBX homeobox 3	GL018699	1.69
Hyper	ENSOCUG00000014169	Ankyrin repeat and SOCS box containing 7	GL018753	1.76
Hyper	ENSOCUG00000029446	Tripartite motif containing 60	GL018701	1.79
Hyper	ENSOCUG00000012963	Suppressor of cancer cell invasion	GL018699	1.81
Hyper	ENSOCUG00000006503	TBC1 domain family member 2B	GL018746	1.86
Hyper	ENSOCUG00000023002	Kelch-like protein 20	GL018825	1.90
Hyper	ENSOCUG00000001522	SH3 domain binding glutamate rich protein	GL018907	1.91
Hyper	ENSOCUG00000000146	IQ motif containing gtpase activating protein 1	GL018738	1.92
Hyper	ENSOCUG00000007308	Zinc finger and SCAN domain containing 2	GL018738	1.92
Hyper	ENSOCUG00000011791	Receptor interacting serine/threonine kinase 1	GL018715	1.93
Hyper	ENSOCUG00000025866	Adenylate kinase 8	GL018764	1.97
Hyper	ENSOCUG00000007599	Nuclear receptor subfamily 6 group A member 1	GL018699	1.98
Hyper	ENSOCUG00000009825	Biphenyl hydrolase like	GL018715	2.03
Hyper	ENSOCUG00000014888	Interferon alpha and beta receptor subunit 1	GL018729	2.08

Hyper	ENSOCUG00000016878	BRCA2, DNA repair associated	GL018702	2.11
Hyper	ENSOCUG00000021019	Ras protein specific guanine nucleotide releasing factor 1	GL018746	2.27
Hyper	ENSOCUG00000014818	Potassium calcium-activated channel subfamily N member 2	GL019132	2.28
Hyper	ENSOCUG00000005747	Collagen type XVI alpha 1 chain	GL018704	2.28
Hyper	ENSOCUG00000012670	Arf-GAP with gtpase, ANK repeat and PH domain-containing protein 1	GL018948	2.37
Hyper	ENSOCUG00000016499	Nad	GL018715	2.38
Hyper	ENSOCUG00000008082	Gtpase activating Rap/rangap domain like 3	GL018699	2.39
Hyper	ENSOCUG00000029680	Putative alpha-1-antitrypsin-related protein	GL018883	2.46
Hyper	ENSOCUG00000016865	Zinc finger protein 831	GL018755	2.52
Hyper	ENSOCUG00000003587	Zinc finger MIZ-type containing 1	GL018836	2.52
Hyper	ENSOCUG00000013908	Lysine acetyltransferase 5	GL019220	2.59
Hyper	ENSOCUG00000001607	Putative serine protease 41	GL018828	2.61
Hyper	ENSOCUG00000002047	Transient receptor potential cation channel subfamily M member 8	GL018736	2.62
Hyper	ENSOCUG00000011025	Contactin 4	GL018703	2.63
Hyper	ENSOCUG00000015227	ADAM metallopeptidase with thrombospondin type 1 motif 17	GL018753	2.64
Hyper	ENSOCUG00000010830	Proline rich coiled-coil 2B	GL018764	2.76
Hyper	ENSOCUG00000006729	G protein-coupled receptor kinase 3	GL018766	2.78
Hyper	ENSOCUG00000024741	Transmembrane protease, serine 2	GL019053	2.80
Hyper	ENSOCUG00000014362	Integrin subunit alpha L	GL018752	2.83
Hyper	ENSOCUG00000026490	Arylacetamide deacetylase-like 4	GL018739	2.92
Hyper	ENSOCUG00000013433	Slingshot protein phosphatase 1	GL018777	3.05
Hyper	ENSOCUG00000015765	Isocitrate dehydrogenase 3	GL018746	3.09
Hyper	ENSOCUG00000003258	Cytochrome c oxidase subunit 5A, mitochondrial	GL018768	3.15
Hyper	ENSOCUG00000003266	SIN3 transcription regulator family member A	GL018768	3.15
Hyper	ENSOCUG00000016234	SV2 related protein	GL018777	3.25
Hyper	ENSOCUG00000015510	C2 calcium dependent domain containing 2	GL019181	3.40
Hyper	ENSOCUG00000012944	Ubiquitin conjugating enzyme E2 G2	GL019021	3.66
Hyper	ENSOCUG00000023837	PR domain zinc finger protein 15		4.75

Δβ: Methylation difference calculated as mean MOVET DNA methylation minus mean NC DNA methylation.
