# Supplementary material for: Early Embryo Exposure to Assisted Reproductive Manipulation Induced Subtle Changes in Liver Epigenetics with No Apparent Negative Health Consequences in Rabbit
Source: Int J Mol Sci. 2021 Sep 8;22(18):9716. doi: 10.3390/ijms22189716 (PMC8467347; doi:10.3390/ijms22189716)
Supplement: Supplementary file 1 [file ijms-22-09716-s001.zip › Supplementary Table S5.pdf]

Supplementary Table S5. Functional analysis of differentially methylated genes in liver tissue of F3 generation animals derived from early embryos exposed to the combination of multiple ovulations, vitrification and embryo transfer (MOVET) and naturally-conceived (NC).

Type+	Category*	Term	Count	p-value
Hypo	BP	Regulation of apoptotic process	2	0.09
Hypo	CC	Cell cortex	2	0.08
Hypo	CC	Recycling endosome	2	0.09
Hypo	KEGG	Endocytosis	3	0.04
Hypo	KEGG	Glycerolipid metabolism	2	0.09
Hyper	BP	DNA damage response, signal transduction by p53 class mediator resulting in transcription of p21 class mediator	2	0.03
Hyper	BP	Cell aging	2	0.04
Hyper	BP	Positive regulation of transcription from RNA polymerase II promoter	5	0.04
Hyper	BP	Response to gamma radiation	2	0.06
Hyper	BP	Intrinsic apoptotic signalling pathway in response to endoplasmic reticulum stress	2	0.06
Hyper	BP	Establishment of protein localization	2	0.07
Hyper	CC	Transcription factor complex	4	0.01
Hyper	CC	Cytosol	7	0.01
Hyper	CC	Growth cone	2	0.08
Hyper	KEGG	Huntington's disease	4	0.03
Hyper	KEGG	Natural killer cell mediated cytotoxicity	3	0.05
Hyper	KEGG	Transcriptional misregulation in cancer	3	0.10

+ Type is referred to the epigenetic status of the gene: hypomethylated (hypo) or hypermethylated (hyper). *Functional analysis was referred to the GO term annotation according to the biological process (BP), cellular component (CC) and molecular function (MF) classification, and the KEGG pathways in which they are involved. Red denotes statistical differences at $p<0.05$.
